# Extrasphincteric anal fistula with gluteal extension: A case report and literature review

**DOI:** 10.1097/MD.0000000000045818

**Published:** 2025-11-14

**Authors:** Jingxing Chen, Wei Peng, Dengming Yu, Qian Liang, Junwei Chen

**Affiliations:** a Anorectal Department, The Third People’s Hospital of Longgang, Clinical Institute of Shantou University Medical College (The Third People’s Hospital of Longgang District Shenzhen), China; b Department of Traditional Chinese Medicine Diagnosis, Guangzhou University of Chinese Medicine, China; c Rehabilitation Medicine Department, The Third People’s Hospital of Longgang, Clinical Institute of Shantou University Medical College (The Third People’s Hospital of Longgang District Shenzhen), China.

**Keywords:** buttock, extrasphincteric anal fistula, infection, non-colitis-associated

## Abstract

**Rationale:**

Anal fistulas typically result from infected anal glands, presenting with symptoms like recurrent purulent discharge, pain, and itching around the anus. Infections are commonly located in the skin, soft tissues, or spaces surrounding the rectum and anal canal. Notably, extensive subcutaneous tissue infections in the buttocks are exceedingly uncommon. Distinguishing this condition from anal fistulas associated with Crohn disease poses a significant diagnostic challenge.

**Patient concerns:**

A 27-year-old female patient presented with pain, swelling, and purulent discharge in the buttocks persisting for 6 months, with a recent exacerbation over the last 2 days.

**Diagnoses:**

The diagnoses were established through a comprehensive medical history assessment, physical examination, enhanced magnetic resonance imaging of the anal canal, and electronic colonoscopy, revealing: complex anal fistula, infected sinus in the buttock area, constipation, and mild anemia.

**Interventions:**

The surgical intervention involving complete fistula dissection, excision of the affected area’s apex in the buttocks, removal of necrotic material, fibrous tissue debridement, and thread-draped drainage facilitated fistula tract healing and markedly enhanced patients’ quality of life.

**Outcomes:**

After treatment, the patient showed no purulent discharge, swelling, or pain in the buttocks, and there was no bleeding or fluid leakage. The patient was followed up for 18 months after the treatment, and the wound healed well, with a satisfactory treatment outcome.

**Lessons:**

In the context of diagnosing and treating anal fistulas, it is crucial to abandon the notion that infection is limited to the perianal region. Utilizing advanced magnetic resonance imaging of the anal canal is essential for precise localization, while employing a combination of diagnostic examinations is warranted for accurate differential diagnosis. Postsurgical follow-up of anal fistula patients for a minimum of 1 year is advisable to monitor recurrence and address predisposing factors like constipation. In instances of uncommon variants, it is advisable to engage in multidisciplinary cooperation to synthesize insights from various specialties, thereby enhancing the scientific rigor of diagnostic and therapeutic approaches.

## 1. Introduction

Anal fistula is a common anorectal condition characterized by a chronic granulomatous infection that develops following perianal infections. In these instances, the discharge of pus is obstructed from flowing into the anal canal, instead spreading through the interstitial spaces between the sphincters. This persistence occurs even after drainage or spontaneous rupture, leading to the formation of an external opening.^[1]^ The incidence rate of anal fistula is reported to be 8.6 per 100,000 individuals, with a higher prevalence among men compared to women, with ratios varying from 1.8:1 to 6.6:1 among different populations.^[2]^ Primarily, anal fistulas are associated with the cryptoglandular theory, with approximately 80% originating from anorectal infections. Moreover, it is noted that 30–50% of patients with perianal abscesses progress to develop fistulas.^[2]^

Anal fistulas are commonly classified based on their relationship with the anal sphincter according to Parks’ classification. The classification includes 4 types: Intersphincteric fistula: the fistula tract passes through the internal anal sphincter. Transsphincteric fistula: The tract progresses from the internal sphincter to the anal margin, situated between the deep and superficial parts of the external sphincter, indicating both high and low fistulas. Suprasphincteric fistula: The tract ascends above the anal rectal ring, categorized as a high fistula. Extrasphincteric fistula: The tract extends beyond the anal rectal ring, possibly due to drainage from the pelvic rectal space or underlying conditions such as cancer or Crohn disease. This type typically has its internal opening located outside the anal sinus area.^[3–5]^Studies have indicated the incidence of these fistulas in a cohort of 419 patients who underwent continuous surgery over a 2-year period: intersphincter fistulas – 10% (42/419), intraperhincter fistulas – 9.5% (40/419), suprasphincter fistulas – 5.5% (23/419), and extraperhincter fistulas – 0%.^[6]^

The precise pathogenesis of extrasphincteric anal fistula remains uncertain, although its development has been linked to elevated levels of tumor necrosis factor α, transforming growth factor β, and IL-13 cytokines. These factors facilitate epithelial mesenchymal transition and cellular invasion, culminating in epithelial-to-mesenchymal transition, tissue restructuring, and fistula genesis. Various epithelial-to-mesenchymal transition inducers in Crohn disease fistula tissue have been identified by researchers and subsequently confirmed through in vitro models.^[7,8]^ Subcutaneous infections in the buttock typically originate from local injections, trauma, or conditions such as carbuncles, furuncles in individuals with diabetes, or hidradenitis suppurativa. Nevertheless, extrasphincteric anal fistulas accompanied by extensive buttock infections are exceptionally uncommon. On January 16, 2024, our team admitted a patient with a non-colitis-associated extrasphincteric complex anal fistula that resulted in a substantial subcutaneous infection in the buttock region, leading to anemia. Following appropriate treatment, the patient exhibited marked improvement, and this case is detailed herein.

## 2. Case presentation

A 27-year-old female was admitted to the Third People’s Hospital of Longgang District, Shenzhen, on January 16, 2024, with worsening buttock swelling, pain, and purulent discharge for 6 months, intensifying over the past 2 days. Initially experiencing anal pain 6 months prior, she self-medicated with an unspecified drug for 1 month without relief. Three months ago, the pain extended from the anus to the buttocks, leading to significant swelling and pus discharge, prompting hospital admission. She had not received any surgical treatment before admission. Her body mass index was 20 kg/m², and she reported a 2-year history of constipation and hard stools, with bowel movements twice weekly. There was no history of coronary heart disease, diabetes, hypertension, trauma, blood transfusions, or surgeries. She denied smoking, alcohol use, or harmful habits, and there was no family history of genetic diseases.

**Figure 1.**
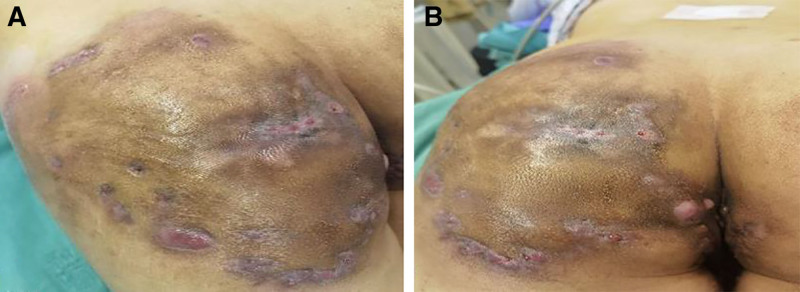
(A) The location and size of the infection; (B) hip infection is associated with anal fistula.

Physical examination upon admission: Body temperature 36.4℃; pulse 105 beats per minute; respiration 20 breaths per minute; blood pressure 129/78 mm Hg. The patient was conscious, with a tired mental state, lack of strength and reluctance to speak, and a soft voice. No obvious abnormalities were found in the physical examination of the heart, lungs, and abdomen. Neither the liver nor the spleen was palpable under the costal margin. The hepatic dullness boundary was normal, there was no shifting dullness, and there was no tenderness on percussion of the renal area. Special examination findings: Extensive skin pigmentation, bridged scars, and multiple ulcer openings were observed in the perianal and perineal areas and both buttocks (the lesion area on the left thigh was larger than that on the right). The subcutaneous fistulas were fused and interconnected, and there were a total of 22 ulcer openings. Palpation of the lesions revealed an obvious fluctuating sensation and significant tenderness. When pressed, grayish-red purulent and bloody fluid flowed out from the ulcer openings. The pus was thick and foul-smelling. A cord-like structure extending towards the anus could be felt in the left buttock lesion. The infected area of the entire buttock accounted for approximately 3% of the body surface area. Digital rectal examination: A hard nodule and a cord-like structure leading to and connected with the left buttock lesion area could be felt at the rectal segment at the 3 o’clock position in the lithotomy position, about 5 cm from the anus. There was no tenderness in the anal sinus area at the 3 o’clock position. A cord-like structure could be felt at the 6 o’clock position leading to the anal sinus area at the 9 o’clock position and extending along the skin to the subcutaneous tissue of the right buttock at the 9 o’clock position as shown in Figure 1A and B.

**Figure 2.**
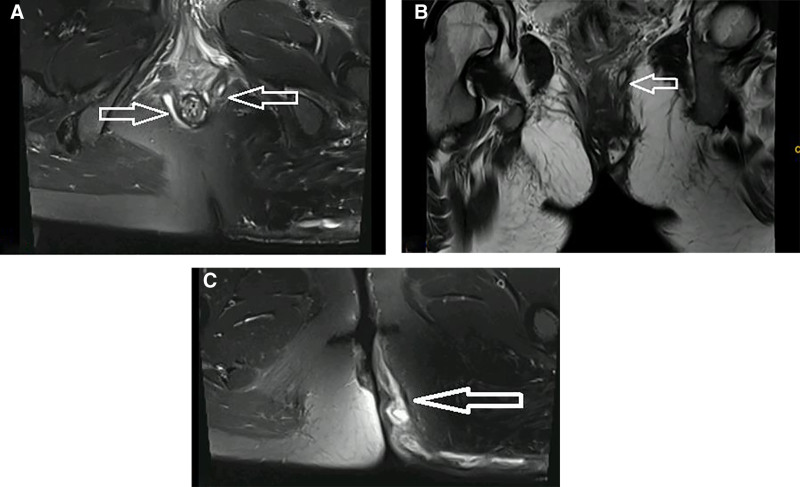
(A) Fistula direction; (B) the internal orifice of the left fistula was located in the adnexa of the levator anal muscle; (C) spread of infection to the subcutaneous area of the buttocks.

Auxiliary inspection: January 16, 2024 Emergency blood routine + reticulin + high-sensitivity C-reactive protein (hs-CRP): hemoglobin 94 (g/L)↓ (normal range 115–150g/L), platelet count 360 (10^9^/L) (Normal range 125–350g/L), hematocrit (HCT) 0.31%(Normal range 0.35–0.45%), WBC count 11.1 (10^9^/L) (normal range 3.5–9.5 10^9^/L), hs-CRP 74.2 (mg/L); normal range 0.0–10.0 mg/L); electrolytes, random glucose, liver function, renal function, glycosylated hemoglobin, fasting blood glucose, chest radiograph, ECG were normal, hepatitis B 5 items and human immunodeficiency virus antibody were negative, anal canal enhanced NMR: At lithotomy position, a strip of high signal intensity of fat suppression was seen at 3 o’clock of anal canal about 41mm away from anal edge on T2WI, upward to rectum, and outward penetrating internal and external sphincter, and left anterior-medial downward along the outer edge of sphincter to subcutaneous of left buttock. DWI (b = 1000) showed high signal intensity, ADC showed low signal intensity, among which the boundary between left anterior fistula and adjacent levator ani muscle was unclear; enhanced scan showed obvious annular enhancement at the edge, but no enhancement in the center. A strip of high signal intensity of fat suppression was seen at 6 o’clock and 9 o’clock of anal canal about 33mm away from anal margin on T2WI, and it penetrated the internal and external sphincter outwards. It walked to the right antero-medial subcutaneous along the outer edge of sphincter. DWI (b = 1000) showed high signal intensity and ADC showed low signal intensity. There was no abnormality in the shape and signal of puborectal muscle and right levator ani muscle. No abnormality in bilateral ischial rectal fossa (see Fig. 2A–C).

**Figure 3.**
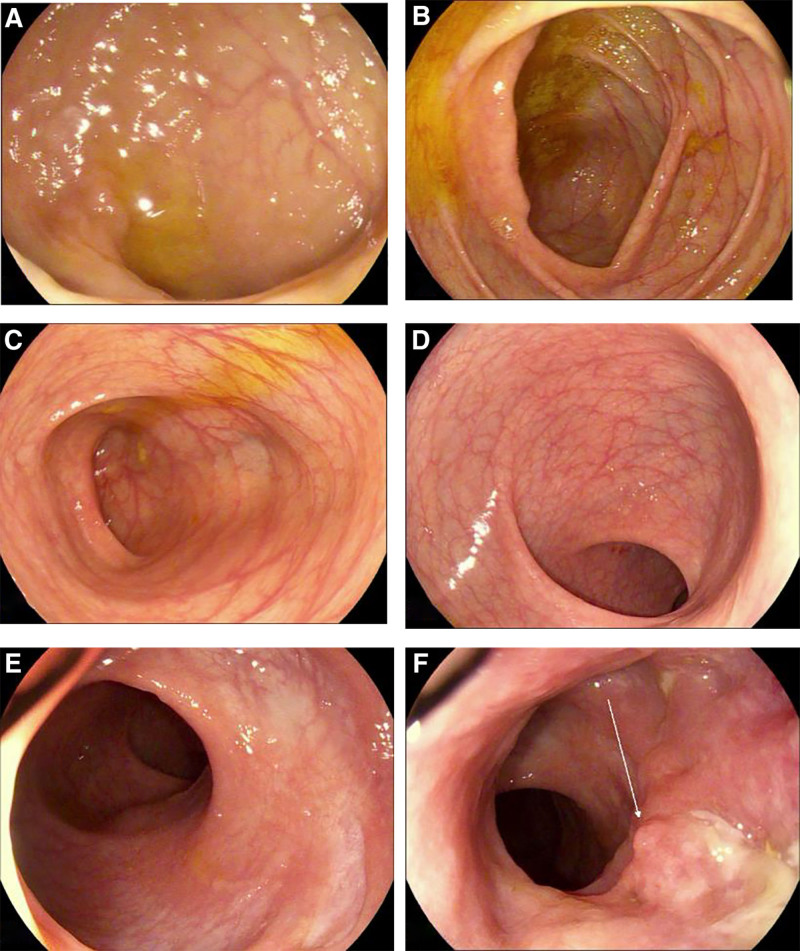
(A) to (E) the complete images of the mucosa under electronic enteroscopy; (F) intestinal opening of anal fistula.

A 6 mm polypoid lesion was identified in the rectum, 4.5 cm from the anal verge, at the 3 o’clock position. The polyp was classified as a type 0-Is lesion and exhibited a NICE type 1 appearance when viewed with flexible spectral imaging color enhancement (FICE). Examination of the sigmoid colon, descending colon, splenic flexure, transverse colon, hepatic flexure, and ileocecal region revealed a smooth mucosal surface, regular haustral folds, and distinct submucosal vascular patterns, without any evidence of erosion, ulceration, or mass lesions. These endoscopic findings were consistent with a hyperplastic rectal polyp, as depicted in Figure 3A to E.

Differential diagnosis includes: Perianal folliculitis and skin furuncle, characterized by localized redness and pain, progressing to swelling with pus formation and shallow lesions not connected to the anus, can be excluded. Anoperineal acute necrotizing fasciitis, a bacterial infection causing extensive tissue necrosis around the anus, perineum, and scrotum, often with fistula formation, rapid onset, and subcutaneous spread, but lacking an internal anal canal opening. Sacroiliac and ischiococcygeal lesions, with slow onset, absence of acute inflammation, thin pus discharge, prolonged healing, and symptoms like appetite loss, low fever, and night sweats, present fistulas distant from the anus without rectal communication, X-rays show bone destruction or hyperplasia. Diagnosis: complex anal fistula (extrasphincteric and transsphincteric types); infectious sinuses in the buttock area; constipation; and mild anemia.

## 3. Treatment process

### 3.1. Surgical procedure

**Figure 4.**
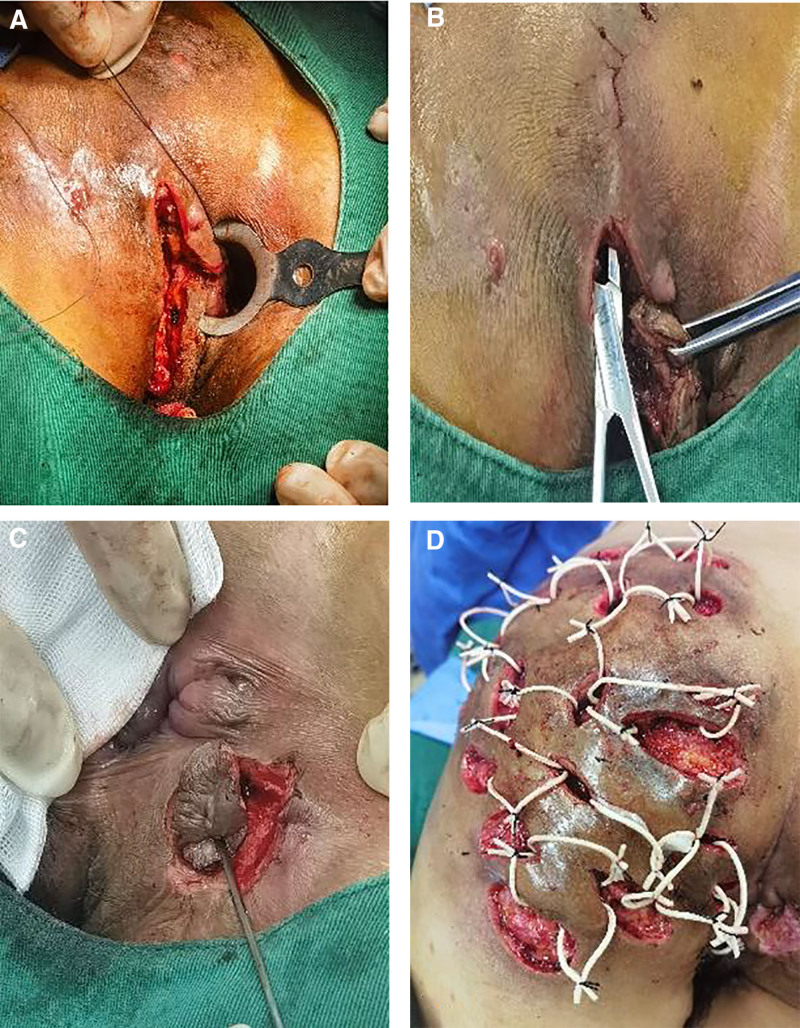
(A) Treatment of the intestinal ostium; (B) probing the direction of infection channels; (C) treatment of right anal fistula; (D) postoperative wiring.

The patient underwent intravenous fluid resuscitation upon admission to the proctology department prior to being transferred to the operating room. There, under subarachnoid anesthesia, a complex surgical intervention was performed. This included the excision of the anal fistula, placement of a seton, modified de-roofing of the buttock fistula tracts, and abscess curettage. Hydrogen peroxide injected at the 3 o’clock lithotomy position external opening caused significant foaming 4 cm above the dentate line in the rectal mucosa, with no anomalies in the anal sinus areas. A similar test at the 9 o’clock position external opening resulted in foaming near the dentate line, confirming the non-interconnection of fistula tracts between the buttocks. A probe inserted at the 3 o’clock position, 3 cm outside the anal margin, traced the fistula through the intersphincteric space to the rectal mucosa 4 cm above the dentate line. An electrosurgical unit, guided by the probe, dissected the fistula tract from the external opening to above the sphincter at the 3 o’clock position near the rectum. The tract was ligated, transected, and the internal opening at the mucosal bulge 4 cm above the dentate line was excised and closed with four 3-absorbable sutures. At the 9 o’clock position, a probe identified a Y-shaped fistula tract extending from the anal sinus to the skin, connecting with another tract from the subcutaneous area to the 6 o’clock anal sinus. Under probe guidance, the fistula tracts and infected areas at the internal openings were excised. The wound edges were shaped into a “V” for optimal drainage. The probe identified a large abscess in the left buttock connected to a high-position anal fistula at the 3 o’clock position, containing several small subcutaneous abscesses. These were incised using an electrosurgical unit for decompression and cleared of fibrous tissue. Cleaning of the abscess cavities exposed significant necrotic tissue, and the wounds were irrigated with hydrogen peroxide, povidone-iodine, and saline. Rubber bands were inserted for loose seton drainage, as shown in Figure 4A to D.

### 3.2. Treatment management

**Figure 5.**
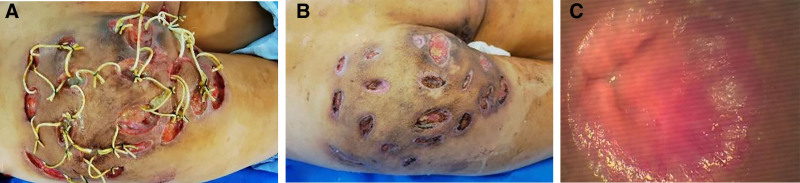
(A) On the 8th day after surgery, the granulation tissue was obviously hyperplasia and the drainage was smooth; (B) the hanging line was removed on the 30th day after surgery; (C) the internal orifice of the intestinal cavity examined on the 30th day after surgery, and it was found that the internal orifice was closed.

During surgery, a pus sample was cultured, identifying both Gram-positive and Gram-negative bacteria, with no detection of Mycobacterium tuberculosis. Postoperative care included intravenous cefuroxime axetil (1.5 g every 12 hours) for 7 days. The wound was cleaned twice daily with hydrogen peroxide and povidone-iodine, and a potassium permanganate sitz bath was administered daily. Anemia was treated with specific medications, and lactulose was used to relieve constipation. By the 8th day postsurgery, granulation tissue began to proliferate at the wound site. The initial postoperative complete blood count showed: hemoglobin 91 g/L (↓), HCT .301, white blood cell count 11.1 × 10^9^/L, and hs-CRP 74.2 mg/L. On day 30 postsurgery, the seton was removed, revealing significant granulation tissue growth and closure of the internal opening, as illustrated in Figure 5A to C.

**Figure 6.**
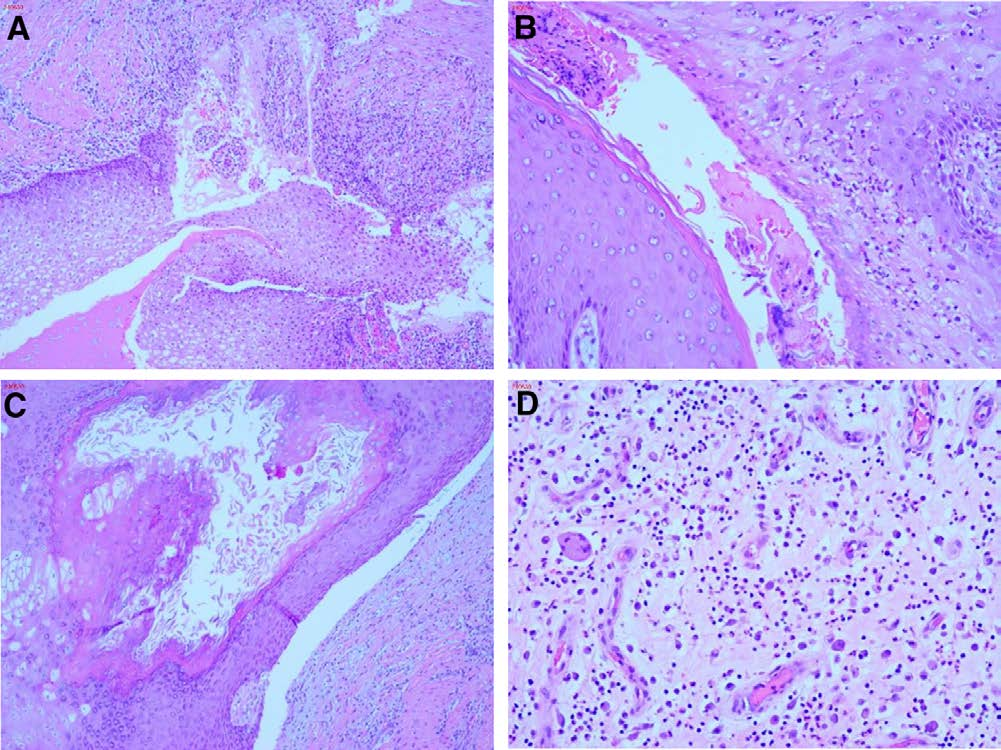
(A to D) Consistent with anal fistula and abscess formation.

The second postoperative complete blood count showed hemoglobin at 96 g/L (↓), HCT at .307, white blood cell count at 6.4 × 10^9^/L, and hs-CRP at 13.7 mg/L. Histopathological analysis of necrotic tissue from the left buttock revealed fragmented skin with dermal cyst-like structures lined by stratified squamous epithelium, without cellular atypia. The cysts contained amorphous keratinized material, alongside localized inflammatory necrosis and granulation tissue. Clusters of necrotic neutrophils were surrounded by proliferative fibrous tissue, as shown in Figure 6A to D.

Following an extensive review of the tests and examination results, the patient was diagnosed with: 1. Non-colitis-associated extrasphincteric anal fistula; 2. Infectious sinuses in the buttock area; 3. Constipation; 4. Mild anemia.

## 4. Treatment outcome

Postoperatively, the patient received antibiotic treatment to prevent infection, iron and folic acid supplements to alleviate anemia symptoms, and lactulose to soften stools and potassium permanganate for sitz baths. Daily monitoring of the wound exudate showed no abnormal redness, swelling, abscess or fluid accumulation. Granulation tissue began to form on the 8th day after the operation, and the drainage tube was gradually removed by the thirtieth day. Throughout the healing process, there was no redness or exudate at the wound site. After 21 days of hospitalization, the patient was discharged in good condition. After 18 months of follow-up, the results showed excellent wound healing, no recurrence of anal fistula, and good healing of the buttocks.

## 5. Discussion

We present a case of a non-colitis-associated extrasphincteric anal fistula with extensive buttock infection, a scenario rarely documented in existing literature. The surgical approach employed here offers an improved method with notable reference value. This case highlights that the infection pathway of anal fistulas extends beyond the perianal space to the subcutaneous region of the buttocks, leading to severe infection. We analyze the pathogenesis, susceptibility factors, and treatment plan as follows:

### 5.1. Pathogenesis analysis of anal fistulas

The pathogenesis of anal fistula involves several theories: 1. The anal gland infection theory posits that infection of the anal glands is the primary cause of anal fistula, a view widely embraced by anorectal specialists since its inception. This theory remains a cornerstone in guiding surgical interventions for anal fistulas.^[9,10]^ 2. The central space infection theory suggests that damage to the anal duct epithelium can lead to infection and abscess formation in the central space, eventually resulting in an anal fistula.^[11]^ 3. The immunological factor theory proposes that systemic or local immune dysfunction contributes to the development or recurrence of anal fistulas.^[12–14]^ 4. The influence of sex hormones: High androgen levels in infants and young males may predispose them to anal fistulas by affecting the anal glands. Some research indicates that elevated sex hormone levels in infants and young children lead to abnormal anal gland development,^[15]^ although the precise relationship between sex hormones and anal fistulas requires further investigation.^[16]^5. Influence of Intestinal Microbes: Studies indicate that bacterial infection is unlikely to be the cause of persistent anal fistulas. Instead, intestinal microorganisms significantly contribute to chronic inflammation through specific inflammatory mediators.^[17]^

The author posits that non-colitis-associated extrasphincteric anal fistula in patients is linked to central space infection. Electronic enteroscopy identified an infected internal opening with damaged epithelium as a possible source of infection. Intestinal disease can extend to the central tendon and space through the damaged anal canal, aided by fiber septa. Inflammatory reactions generate anal fistulas in adjacent spaces. The infected opening, situated 5cm from the anus near the anal riser muscle, was contained by muscular barriers, preventing abdominal spread. Muscles like pubococcygeal, puborectal, and iliac coccygeal halted the infection at the top of the external sphincter, infiltrating the sphincter space. Prolonged infection weakened muscles via inflammatory edema, enabling spread to the subcutaneous buttock, potentially causing anal fistula formation and extensive infection.

### 5.2. Analysis of predisposing factors

Anal fistula development and progression are influenced by patient-specific factors (gender, age, smoking, alcohol use, enteritis, diabetes, obesity), lifestyle habits (constipation or diarrhea), surgical risks, and intrinsic fistula characteristics (type, number of tracts, height, location of internal opening).^[18]^Modifiable risk factors play a crucial role in determining the infection’s severity and progression.^[19]^The patient had a body mass index of 20 kg/m² and no history of major health conditions. There was no indication of trauma, surgeries, immunodeficiency disorders, or risky behaviors. She had no smoking, alcohol consumption, or harmful habits, and no family history of genetic diseases. A chest X-ray excluded pulmonary tuberculosis. Her bowel habits met the Rome IV criteria for constipation: 2 weekly bowel movements with hard stools and difficulty defecating for at least 6 months.^[20]^

Constipation is associated with changes in the gut microbiome, primarily composed of the bacterial phyla Firmicutes, Bacteroidetes, Actinobacteria, and Proteobacteria. This microbiome interacts with the host’s intestinal mucosa to regulate immunity, protect against pathogens, assist in digestion, and synthesize vitamins essential for host nutrition.^[21–26]^

Studies consistently show significant differences in gut microbiota composition between constipated individuals and non-constipated counterparts,^[27,28]^ with constipated individuals exhibiting reduced beneficial bacteria (e.g., Lactobacillus, Roseburia, Firmicutes) and increased pathogenic microbes (e.g., atypical Staphylococci, Staphylococcus, Prevotella).^[29,30]^ Bacteroidetes presence in colonic mucosa correlates with constipation severity, while elevated Firmicutes are associated with longer colonic transit times.^[31]^ These imbalances in the gut microbiome due to constipation may negatively impact gut health, promote bacterial translocation, and potentially contribute to extrasphincteric anal fistula development. Psychological counseling should be integrated into constipation treatment, as psychological factors and constipation may interact through the gut-brain axis, affecting psychological states and gastrointestinal functions.^[32]^

### 5.3. Differential diagnosis of subcutaneous infection in the buttock

Extensive subcutaneous infections in the buttock due to anal fistulas are exceedingly rare. Buttock infections are categorized as either superficial, involving subcutaneous tissues, or deep, occurring below the deep fascia. Typical causes include injections, trauma, cysts, or foreign bodies, but those linked to anal fistulas are infrequent.^[33–35]^

The infection was definitively linked to an anal fistula, setting it apart from typical buttock infections. It is crucial to distinguish this condition from hidradenitis suppurativa, a chronic inflammatory skin disorder that can mimic anal fistulas. Hidradenitis suppurativa initially manifests as subcutaneous nodules, which can evolve into abscesses, fistulas, and scarring.^[36–38]^ This condition can affect the perianal region, axillae, groin, and back. Clinicians should carefully review the patient’s history, conduct a digital rectal examination, and differentiate perianal diseases associated with Crohn disease.^[39]^ Consideration should be given to atypical verrucous lesions and colonoscopic signs of Crohn colitis. If Crohn disease is excluded, a colonoscopy is advisable, even in the absence of significant gastrointestinal symptoms.^[40]^

### 5.4. Selection and analysis of relevant treatment options

The choice of surgical method depends on the patient’s specific conditions, with each option offering distinct pros and cons, as detailed in Table 1. For anal fistulas, common procedures include seton placement, fistulotomy, or fistulectomy, alongside sphincter-preserving techniques like video-assisted fistula tract ligation. Emerging methods under study involve stem cell transplantation and laser or radiofrequency ablation of the fistula tract.^[7,41]^ Surgical approaches for buttock infections range from local interventions such as incision and drainage or de-roofing, to lesion excisions like wide infection excision or the STEEP procedure, and wound repair methods including primary closure, flap transplantation, or secondary healing.^[42–46]^ In this case, a multidisciplinary approach was chosen, involving complex fistula excision, seton placement, de-roofing of the buttock fistula, and abscess curettage for several reasons:

1. Excision is recommended for non-inflammatory rectal or colonic fistulas. The approach varies based on location and complexity.^[47]^ A case of non-colitis-associated extrasphincteric anal fistula with extensive buttock infection in a 30-year-old female with preserved sphincter function was managed through thorough excision to reduce recurrence risk. Preservation of the sphincter muscle during excision is crucial. Ligation, excision, and suturing of the internal opening above the dentate line were performed to align with fistula tract ligation principles, reducing the risk of complications. Skilled colorectal surgeons should manage complex fistulas to avoid sphincter damage. Treatment followed American Society of Colon and Rectal Surgeons guidelines,^[41]^ emphasizing sphincter preservation for the low-lying right buttock fistula.
2. In this instance, we employed a modified de-roofing technique for the buttock fistula, which expedited the patient’s recovery and shortened the hospital stay. The conventional de-roofing method involves the excision of only the lesion’s top layer while preserving the base and surrounding fibrous and scar tissues. However, the residual fibrous tissue and concealed sinuses might elevate the risk of postoperative recurrence.^[42,43]^ During the procedure, probes were utilized to delineate each sinus opening and the connected fistula tract. The lesion’s top portion was excised to fully reveal the base, followed by the removal of any gelatinous necrotic material, defining the modified de-roofing approach.^[44]^

A seton technique, involving the insertion of a drainage seton within the fistula tract, is commonly used for complex anal fistulas and abscesses.^[48,49]^ This technique includes cutting seton and loose seton variations, effectively managing costs and drainage. Surgical methods for buttock infections should be selected based on patient-specific factors such as the condition’s stage, affected region, general health, and postexcision site reconstruction importance for postoperative outcomes as shown in Table 2.

conclusion: Complex anal fistulectomy involving complete resection of anal fistulas and intersphincter fistula ligation near the inner orifice and between the inner orifice and the muscular layer was performed to preserve sphincter function while removing infected lesions. This approach has shown a comprehensive cure rate of 76%.^[50]^ Modified decapitation was used for posterior fistulas to prevent recurrence by addressing residual fibrous tissue and cryptic sinus, with a recurrence rate of 14%.^[44]^ These surgical techniques represent improvements with potential reference value.

### 5.5. Analysis of postoperative antibiotic use for anal fistula

The American Society of Colon and Rectal Surgeons’ 2022 clinical guidelines suggest that postoperative antibiotics do not improve cure rates or reduce recurrence in perianal abscesses and fistulas.^[51]^ Antibiotics are recommended for severe cellulitis, immunocompromised patients, or those with systemic conditions. Empirical antibiotic treatment is justified for patients with perianal and buttock infections caused by both Gram-positive and Gram-negative bacteria. Perianal abscesses and fistulas commonly stem from anal gland infections, supporting the rationale for perioperative antibiotic use to prevent further infection and new fistula formation.^[52]^

### 5.6. Analysis and treatment of anemia

Systemic or extensive local infections can trigger a body stress response characterized by increased inflammatory factors and emergency hormone synthesis via specific pathways. This response impairs immune function but boosts red blood cells’ phagocytic capacity, leading to reduced blood cell count.^[53–57]^ Inflammatory factors from immune cells can hinder red blood cell production and erythropoietin synthesis, leading to anemia. Infections like anal fistulas trigger emergency responses, reducing red blood cells.^[58]^ Treating anemia with iron and folic acid aids wound healing. Prompt surgery to halt infection spread is advised, followed by iron and folic acid supplements for wound recovery.

## 6. Conclusion

This case study highlights the importance of accurate diagnosis and differential diagnosis in anal fistula management. It challenges the notion that anal fistula infections are localized to the perianal region and underscores the need for vigilance when patients present with perianal symptoms alongside buttock swelling and purulent discharge. Utilizing enhanced anal canal MRI is recommended to precisely identify the fistula’s location and the extent of infection. Integrating the patient’s medical history, digital rectal examination, colonoscopy, and pus etiological testing is crucial in distinguishing anal fistula-related buttock infections from other differential diagnoses to prevent misdiagnosis. In terms of treatment, a tailored surgical approach is advised based on the patient’s clinical presentation. For complex anal fistulas complicated by buttock infections, a combined strategy involving “complex fistula resection + modified de-roofing + seton drainage” is suggested to address lesion removal while safeguarding sphincter function. Antibiotic therapy should be administered according to infection severity and etiological findings, with concomitant management of complications such as anemia (via iron supplements and folic acid) and constipation (using lactulose). Postoperative care should include proactive follow-up for at least 1 year to monitor wound healing and detect any signs of recurrence. Addressing predisposing factors like constipation through gut microbiota regulation and psychological support is recommended to mitigate recurrence risk. Furthermore, special attention should be given to unique anal fistula cases, such as the non-colitis-related extrasphincteric anal fistula discussed in this study. Emphasizing multidisciplinary collaboration and incorporating input from various specialties can enhance the scientific rigor of diagnosis and treatment, offering valuable insights for similar clinical scenarios.

## 7. Limitation

While this detailed case analysis provides important clinical insights, we acknowledge the need for larger prospective studies. In the future, large-sample and multicenter studies can be conducted.

**Table 1:** Advantages and disadvantages of anal fistula surgeries.

Surgical method	Scope of clearance	Applicable population	Advantages	Disadvantages
Traditional surgery				
Seton placement	–	Complex anal fistula	Simplifies the procedure, safeguards sphincter function, and diminishes fecal incontinence risk	Gradual sphincter sectioning could impair anal functionality
Fistulotomy and fistulectomy	Complete removal or excision of the fistula tract	Fistulas with a mature or external tract	Thoroughly eradicates the lesion	Potential sphincter impairment, elevating postsurgical incontinence risk
Sphincter-preserving surgery				
Fistula tract ligation	Partial ligation and resection were performed on the sphincter fistula, and the remaining part was treated with the tunnel resection method	Fistulas with a mature or external tract	Maintains anal sphincter integrity, streamlines the procedure, minimizes wound size, mitigates tissue damage, and accelerates recovery	Increased recurrence likelihood
Video-assisted fistula excision	Comprehensive excision of the entire tract and sealing of the internal opening	Low or intricate fistulas	Inflicts minimal trauma, and lowers fecal incontinence risk	Potential electrothermal damage to healthy tissue could delay wound healing
Exploration of new techniques				
Stem cell transplantation	Direct stem cell injections into the sutured internal opening or adjacent tissue, and throughout the fistula tract via the external opening	Anal fistulas in Crohn disease	Eliminates the need for tract excision, significantly mitigates anal sphincter damage, and reduces postoperative incontinence probability	Demands advanced technical and equipment capabilities, with an ambiguous recurrence rate
Laser radiofrequency fistula tract closure	Laser-induced obliteration of the fistula tract tissue, facilitating tract contraction and closure	Fistulas with a mature or external tract	Causes minimal trauma, preserves anal functionality, and ensures rapid postoperative recuperation	Less efficacious for extensive, complex fistulas, notably those with larger diameters

**Table 2:** Advantages and disadvantages of buttock surgery options.

Surgical method	Scope of clearance	Applicable population	Advantages	Disadvantages
Local treatment				
Incision and drainage	Single or localized abscess	Buttock furuncles, carbuncles	Straightforward procedure, significantly alleviates pain	Elevated rates of recurrence and infection
De-roofing	The abscess’s apex and sinus, including the internal gelatinous material	Recurrent nodules and sinuses	Expedited postoperative recovery, markedly relieves pain	Elevated rates of recurrence and infection
Modified de-roofing	Beyond de-roofing, includes excision of the lesion’s base and all inter-incisional fibrous tissue	Recurrent nodules and sinuses	Swift recovery, markedly alleviates pain, reduced recurrence rate	Marginally extended healing duration
Lesion excision				
Wide excision	Entire lesion	Individuals with local recurrent surgery, unmanaged infections, and severe widespread infections	Comprehensive debridement, minimal recurrence rate	Prolonged postoperative healing, increased risk of complications
STEEP procedure	Entire affected region and fibrotic tissue	Patients with Hurley Stage II/III hidradenitis suppurativa	Conserves healthy tissue, accelerates postoperative recovery, minimizes postoperative scar contraction	Recurrence rate remains uncertain

## Acknowledgments

We would also like to express our gratitude to all the researchers who participated in this study, including doctors, nurses, as well as the editors and reviewers who provided suggestions for the revision of this article.

## Author contributions

**Conceptualization:** Jingxing Chen, Dengming Yu, Qian Liang.

**Data curation:** Jingxing Chen, Qian Liang.

**Formal analysis:** Jingxing Chen, Qian Liang.

**Funding acquisition:** Jingxing Chen, Qian Liang.

**Investigation:** Jingxing Chen, Wei Peng.

**Methodology:** Jingxing Chen, Wei Peng, Qian Liang.

**Project administration:** Jingxing Chen.

**Resources:** Jingxing Chen, Wei Peng, Dengming Yu.

**Software:** Jingxing Chen, Dengming Yu.

**Supervision:** Jingxing Chen, Wei Peng, Dengming Yu, Junwei Chen.

**Validation:** Jingxing Chen, Junwei Chen.

**Visualization:** Jingxing Chen, Junwei Chen.

**Writing – original draft:** Jingxing Chen, Junwei Chen.

**Writing – review & editing:** Jingxing Chen, Junwei Chen.
